# Impact of signs and symptoms on the prognosis of patients with HFmrEF

**DOI:** 10.1186/s12872-023-03436-z

**Published:** 2023-08-24

**Authors:** Zhican Liu, Yunlong Zhu, Lingling Zhang, Mingxin Wu, Haobo Huang, Ke Peng, Wenjiao Zhao, Sihao Chen, Xin Peng, Na Li, Hui Zhang, Yuying Zhou, Yongliang Chen, Sha Xiao, Liqing Yi, Jie Fan, Jianping Zeng

**Affiliations:** 1 Department of Cardiology, Xiangtan Central Hospital, Xiangtan, 411100 China; 2 Graduate Collaborative Training Base of Xiangtan Central Hospital, Hengyang Medical School, University of South China, Hengyang, 421001 Hunan China; 3 Department of Cardiology, The Second Xiangya Hospital of Central South University, Changsha, 410011 Hunan China; 4 Department of Scientific Research, Xiangtan Central Hospital, Xiangtan, 411100 China

**Keywords:** Signs, Symptoms, Heart failure, HFmrEF

## Abstract

**Background:**

Worsening of heart failure (HF) symptoms is the leading cause of medical contact and hospitalization of patients with mildly reduced ejection fraction (HFmrEF). The prognostic value of signs and symptoms for patients with HFmrEF is currently unclear. This study investigated the prognostic impact of signs and symptoms in HFmrEF patients.

**Methods:**

A Cox proportional risk regression model analyzed the relationship between the number of signs/symptoms and outcomes in 1691 hospitalized HFmrEF patients. Ten significant signs and symptoms were included. Patients were divided into three groups (A: ≤2, B: 3–5, C: ≥6 signs/symptoms). Stratified analysis on male and female patients was performed. The primary endpoint was all-cause mortality, and the secondary outcome was a composite of cardiovascular death and heart failure readmission (CV events) post-discharge.

**Results:**

After a median follow-up of 33 months, all-cause mortality occurred in 457 patients and CV events occurred in 977 patients. Incidence of all-cause mortality was 20.7%, 32.3%* and 49.4%*† in group A, B and C of male patients, (*P < 0.05 vs. A, †P < 0.05 vs. B) and 18.8%, 33.6% and 55.8%* in group A, B and C of female patients. Incidence of CV events was 64.8%, 70.1%* and 87.5%* in group A, B and C of male patients, 61.9%, 75.3%, and 86.1%* in group A, B and C of female patients. Multivariate Cox regression showed older age, renal insufficiency, higher number of signs and symptoms (≥ 3, hazard ratio [HR] 1.317, 95% confidence interval [CI] 1.070–1.621, P = 0.009; ≥6, HR 1.982, 95% CI 1.402–2.801, P < 0.001), myocardial infarction, stroke, faster heart rate on admission, and diabetes were independently associated with all-cause mortality(all P < 0.05). Similarly, higher number of signs and symptoms (≥ 3, HR 1.271, 95% CI 1.119–1.443, P < 0.001; ≥6, HR 1.955, 95% CI 1.524–2.508, P < 0.001), older age, renal insufficiency, atrial fibrillation, and diabetes were independently associated with cardiovascular events (all P < 0.05).

**Conclusions:**

Higher number of symptoms and signs is associated with increased risk of all-cause mortality and CV events in HFmrEF patients. Our results highlight the prognostic importance of careful inquiry on HF symptoms and related physical examination in HFmrEF patients.

**Supplementary Information:**

The online version contains supplementary material available at 10.1186/s12872-023-03436-z.

## Introduction

Chronic heart failure (CHF) is characterized by decline in exercise capacity and dyspnea, serves as a common etiology of repeated hospitalizations [1]. It is estimated that around 26 million people worldwide suffer from chronic heart failure [2]. In China, the prevalence rate of age-standardized heart failure (HF) is 1.10%, and the incidence rate is 275 cases per 100,000 person-years [3]. The China-HF study showed that the mortality rate was 4.1% in hospitalized HF patients [4]. Aging population and advances in the treatment of HF may further increase the number of patients with HF, CHF is also associated with huge financial burden on family and the healthcare system [2].Heart failure is a clinical syndrome, the signs and symptoms of which are primarily due to functional or structural heart disease. These functional or structural diseases can include, but are not limited to, cardiomyopathies, coronary artery disease, hypertension, or valvular heart disease [5].

Nowadays, physicians rely more and more on imaging and laboratory tests results to guide the diagnosis and treatment of HF [6], rational use of these modalities are essential, but adds unavoidably to the financial burden on the healthcare system. Independent of imaging and laboratory tests, previous studies have shown that signs and symptoms, which could be obtained by careful and thorough inquiry and physical examination, can also guide the diagnosis and treatment of patients with HF in an economic way [6, 7]. Besides, information on signs and symptoms is valuable for decision making regarding the need of hospitalization or not [8]. In a post-hoc analysis of the PARAGON-HF trial, more HF signs and symptoms were associated with higher risk of HF hospitalization and cardiovascular death in HF patients with preserved ejection fraction (HFpEF) [9]. A similar risk relationship has been shown in studies examining various combinations of signs and symptoms in patients with reduced ejection fraction (HFrEF) [10–12]. The prognostic value of signs and symptoms in patients with heart failure with mildly reduced ejection fraction (HFmrEF) is currently unclear. In this study, we observed the prognostic impact of signs and symptoms in HFmrEF patients.

## Methods

### Study design and population

**Fig. 1:**
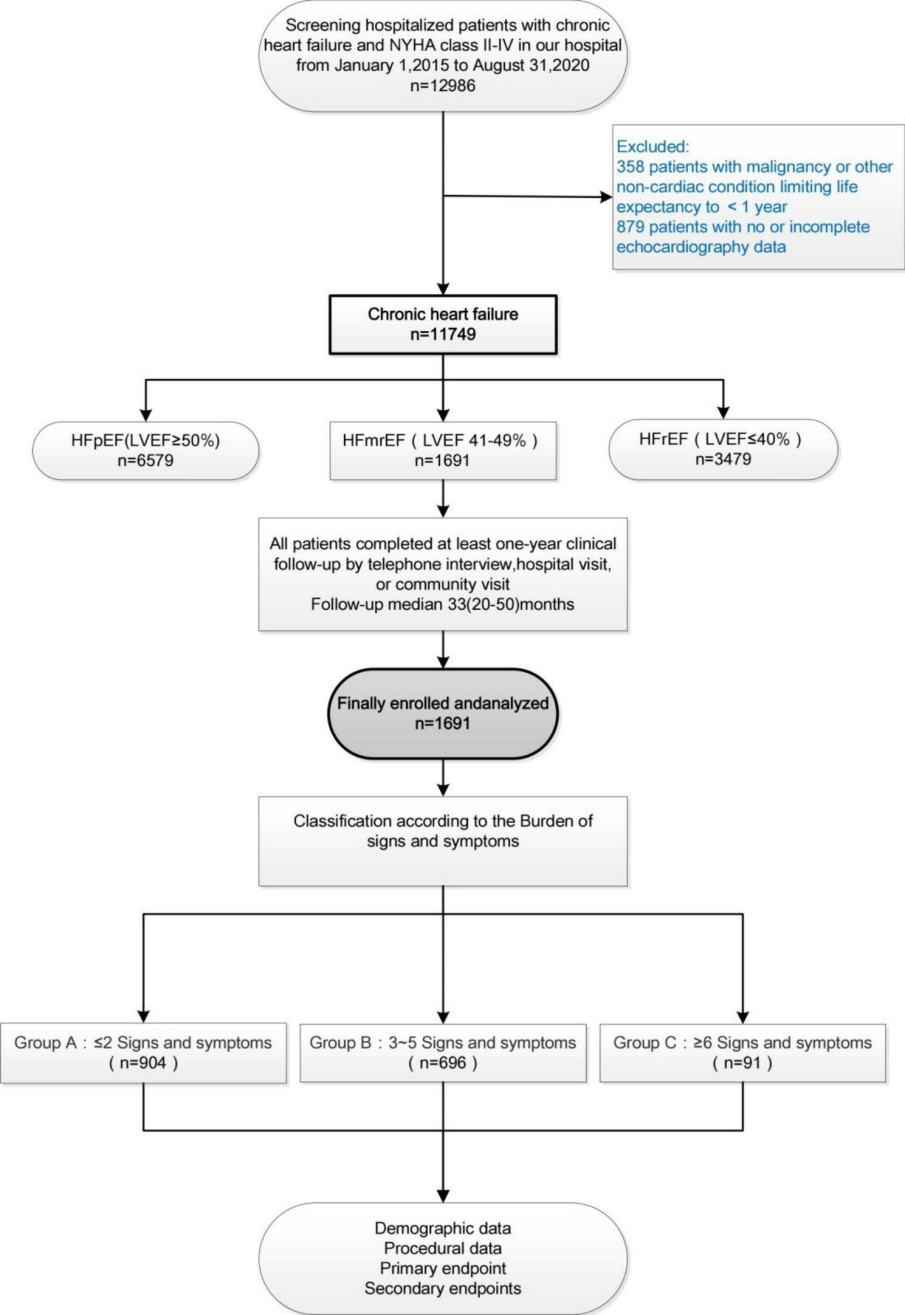
Flow diagram for participant screening, eligibility and analysis

A total of 1691 patients with HFmrEF admitted to our hospital from 1 to 2015 to 31 August 2020 were included. All patients included in this study were diagnosed with heart failure according to standard AHA and ESC guidelines [5, 13]. In our study [14], the inclusion criteria were as follows: 1)hospitalized patients in the cardiology department were clinically diagnosed chronic HF patients with New York Heart Association (NYHA) functional class II, III, or IV, according to the 2012 guidelines of the ESC Working Group; 2) hospitalization echocardiography detected LVEF of 41–49%. Chronic HF was defined if patients had a history of decompensated HF events within the last 12 months prior to admission. Exclusion criteria are malignancy or other non-cardiac diseases with an expected survival time of less than one year. In this study, HFmrEF patients were divided into three groups according to the number of signs and symptoms: A: ≤2 (n = 904); B: 3–5 (n = 696); and C: ≥6 (N = 91) (Fig. 1; Table 2). Since the distribution of HF types and sex varies considerably among HF patients [15], we also conducted a stratified analysis according to sex.

**Table 1:** Baseline Characteristics by Burden of Signs and Symptoms

	Signs and Symptoms		
	≤ 2(n = 904, Group A)	3 ~ 5(n = 696, Group B)	≥ 6(n = 91, Group C)
**Signs and symptoms, N (%)**			
Chest pain	391 (43.3%)	228 (32.8%)*	20 (22.0%)*
Chest suffocation	223 (24.7%)	449 (64.5%)*	83 (91.2%)*†
Dyspnea at rest	146 (16.2%)	558 (80.2%)*	89 (97.8%)*†
Cough	53 (5.9%)	291 (41.8%)*	70 (76.9%)*†
Jugular venous distension	26 (2.9%)	109 (15.7%)*	53 (58.2%)*†
Hepatojugular reflux sign	0 (0.0%)	3 (0.4%)	8 (8.8%)*†
Rales	119 (13.2%)	458 (65.8%)*	86 (94.5%)*†
Edema	52 (5.8%)	277 (39.8%)*	81 (89.0%)*†
Ascites	5 (0.6%)	24 (3.4%)*	17 (18.7%)*†
Pleural effusion	64 (7.1%)	225 (32.3%)*	71 (78.0%)*†
**Demographics**			
Age, years	66.7 ± 12.3	69.8 ± 12.3*	70.8 ± 11.8*
Female, N (%)	298 (33.0%)	250 (35.9%)	48 (52.7%)*†
Body mass index, kg/m2	25.1 ± 4.2	25.0 ± 4.0	25.4 ± 4.3
Heart rate, bpm	81.0 ± 18.0	86.6 ± 20.7*	93.8 ± 23.7*†
Systolic blood pressure, mmHg	133.4 ± 24.0	139.1 ± 27.5*	146.1 ± 27.0*†
NYHA III + IV, N (%)	425 (47.0%)	464 (66.7%)*	83 (91.2%)*†
**Medical history, N (%)**			
Current smoker	289 (32.0%)	233 (33.5%)	22 (24.2%)
Current drinker	86 (9.5%)	55 (7.9%)	6 (6.6%)
Coronary heart disease	729 (80.6%)	525 (75.4%)*	69 (75.8%)
Hypertension	606 (67.0%)	488 (70.1%)	68 (74.7%)
Hyperlipidemia	218 (24.1%)	114 (16.4%)*	18 (19.8%)
Atrial fibrillation	120 (13.3%)	151 (21.7%)*	25 (27.5%)*
Diabetes	269 (29.8%)	245 (35.2%)	40 (44.0%)*
Stroke	125 (13.8%)	72 (10.3%)	10 (11.0%)
Myocardial infarction	521 (57.6%)	316 (45.4%)*	36 (39.6%)*
COPD	72 (8.0%)	120 (17.2%)*	17 (18.7%)*
Renal insufficiency	164 (18.1%)	206 (29.6%)*	37 (40.7%)*†
**Treatment, N (%)**			
Beta-blocker	707 (78.2%)	569 (81.8%)	74 (81.3%)
ACEi	496 (54.9%)	325 (46.7%)*	38 (41.8%)*
ARB	209 (23.1%)	205 (29.5%)*	38 (41.8%)*†
ARNI	39 (4.3%)	36 (5.2%)	4 (4.4%)
SGLT2i	2 (0.2%)	7 (1.0%)	0 (0.0%)
Diuretics	342 (37.8%)	484 (69.5%)*	69 (75.8%)*
Statins	748 (82.7%)	597 (85.8%)	77 (84.6%)
Spironolactone	374(41.4%)	362(52.0%)	39(42.9%)
Digoxin	21(2.3%)	33(4.7%)*	10(11.0%)*†
Antiplatelet Drugs	731 (80.9%)	588 (84.5%)	75 (82.4%)
Positive inotropic drugs	28 (3.1%)	56 (8.0%)*	18 (19.8%)*†
Vasoactive drugs	23 (2.5%)	42 (6.0%)*	5 (5.5%)
PCI	353 (39.0%)	199 (28.6%)*	13 (14.3%)*†
**Serology**			
NT-proBNP, pg/ml	4439.1 ± 7282.3	8740.2 ± 10759.2*	14542.4 ± 11861.8*†
Hemoglobin, g/dL	126.2 ± 21.8	118.9 ± 23.6*	108.5 ± 23.3*†
Sodium, mmol/L	139.3 ± 3.5	139.4 ± 3.7	139.8 ± 3.7
Uric Acid,µmol/L	345.3 ± 110.6	382.2 ± 119.8*	406.5 ± 136.7*
eGFR, ml/min/1.73 m2	75.7 ± 34.0	64.1 ± 35.5*	49.5 ± 33.6*†
Low density lipoprotein, mmol/L	2.5 ± 1.0	2.4 ± 0.9*	2.4 ± 1.1
**Echocardiography**			
LVEF, %	44.6 ± 2.8	44.3 ± 2.7	44.3 ± 2.6
LAs (mm)	38.0 ± 5.7	40.7 ± 6.5*	41.6 ± 5.3*
LVd (mm)	53.1 ± 6.3	55.1 ± 7.7*	54.9 ± 5.5*
RAs (mm)	36.6 ± 5.3	38.7 ± 6.7*	39.7 ± 8.7*
RVd (mm)	20.6 ± 5.2	21.2 ± 5.3	20.9 ± 5.6
E/e′	14.6 ± 7.1	16.7 ± 8.1*	19.6 ± 8.4*†
PASP(mmHg)	29.4 ± 15.0	36.1 ± 19.6*	41.2 ± 15.8*†

The population was classified according by Burden of Signs and Symptoms. Values for continuous variables are given as means ± SD. Bold represent significant values (p < 0.05).Group A: ≤2 Signs and symptoms; Group B: 3 ~ 5 Signs and symptoms; Group C: ≥6 Signs and symptoms.

*P < 0.05 vs. Group A

†P < 0.05 vs. Group B

Abbreviations: LVEF: left ventricular ejection fraction; NYHA: New York Heart Association Classification; COPD :chronic

obstructive pulmoriary disease; ACEi: angiotensin-converting enzyme inhibitor; ARB: angiotensin receptor blocker; ARNI: angiotensin receptor neprilysin inhibitor; SGLT2i:sodium-dependent glucose transporters 2 inhibitor; NT-proBNP: N-terminal pro-B type natriuretic peptide; eGFR: estimated glomerular filtration rate; PCI: percutaneous coronary intervention; LAs: Left atrial; LVd: left ventricle dimension; RAs :right atrial ; RVd : right ventricle dimension; E/e′: ratio of early transmitral flow velocity to early mitral annular velocity; PASP: pulmonary artery systolic pressure

### Procedures and clinical endpoints

Demographic and procedural data of enrolled patients were collected from hospital charts or databases. All patients were followed up until August 31, 2021. The ten signs and symptoms included in the analysis were as follows: chest pain, chest suffocation, dyspnea, cough, jugular venous distension, hepatojugular reflux sign, rales, edema, ascites, and pleural effusion. The signs and symptoms utilized in our study were primarily based on the physical examinations performed by practicing clinicians, rather than derived from imaging or laboratory tests. Outcome events were obtained by examining hospital records and during follow-up by clinical visit, telephone interviews and community visits. The follow up team consisted seven experienced physicians and nurses. The primary outcome was all-cause death after hospital discharge. The secondary outcome was the composite endpoint of cardiovascular death and heart failure readmission (CV events). Cardiovascular death is death from any cardiovascular mechanism: death from acute myocardial infarction, sudden cardiac death, death from heart failure, death from stroke, death from cardiovascular surgery, death from cardiovascular hemorrhage, and death from other cardiovascular causes.

### Statistical analysis

Cox proportional risk regression models was used to examine the relationship between signs and symptoms and the incidence of all-cause mortality. Number of signs and symptoms were analyzed as a continuous variable (impact of each increase of signs and symptoms on outcome) and as a categorical variable (≤ 2, 3–5, ≥ 6). We adjusted the following baseline covariates: age, systolic blood pressure, heart rate, New York Heart Association functional classification, coronary heart disease, hyperlipidemia, atrial fibrillation, diabetes, myocardial infarction, renal insufficiency, hypertension, stroke, current smokers and current alcohol drinkers.

Data are presented as mean ± standard deviation for normal distributions, median (interquartile range) for skewed distributions, and frequencies (percentages) for categorical variables. Comparisons between the two groups were made using t-tests for continuous measures and Chi-square tests for categorical variables to compare clinical characteristics between groups. Comparisons between the three groups were made using the ANOVA test, and Tamhane’s T2 test was chosen when the variance was not uniform.The Kaplan-Meier method was used to estimate the incidence of cumulative events. Curve fitting was applied to find inflection points. P-values were obtained using the Kruskal Wallis rank sum test for continuous variables and the Fisher exact probability test for count variables. Results were considered significant when p < 0.05. Statistical analyses were performed using a combination of R (http://www.R-project.org) and EmpowerStats software (www.empowerstats.com, X&Y solutions, Inc. Boston MA).

## Results

### Clinical profiles

In this cohort, there were 904 (53.46%) patients with symptom and signs ≤ 2 (group A), 696 (41.16%) patients with symptom and signs 3 ~ 5 (group B) and 91 (5.38%) patients with symptom and signs ≥ 6 (group C, Table 2).

**Fig. 2:**
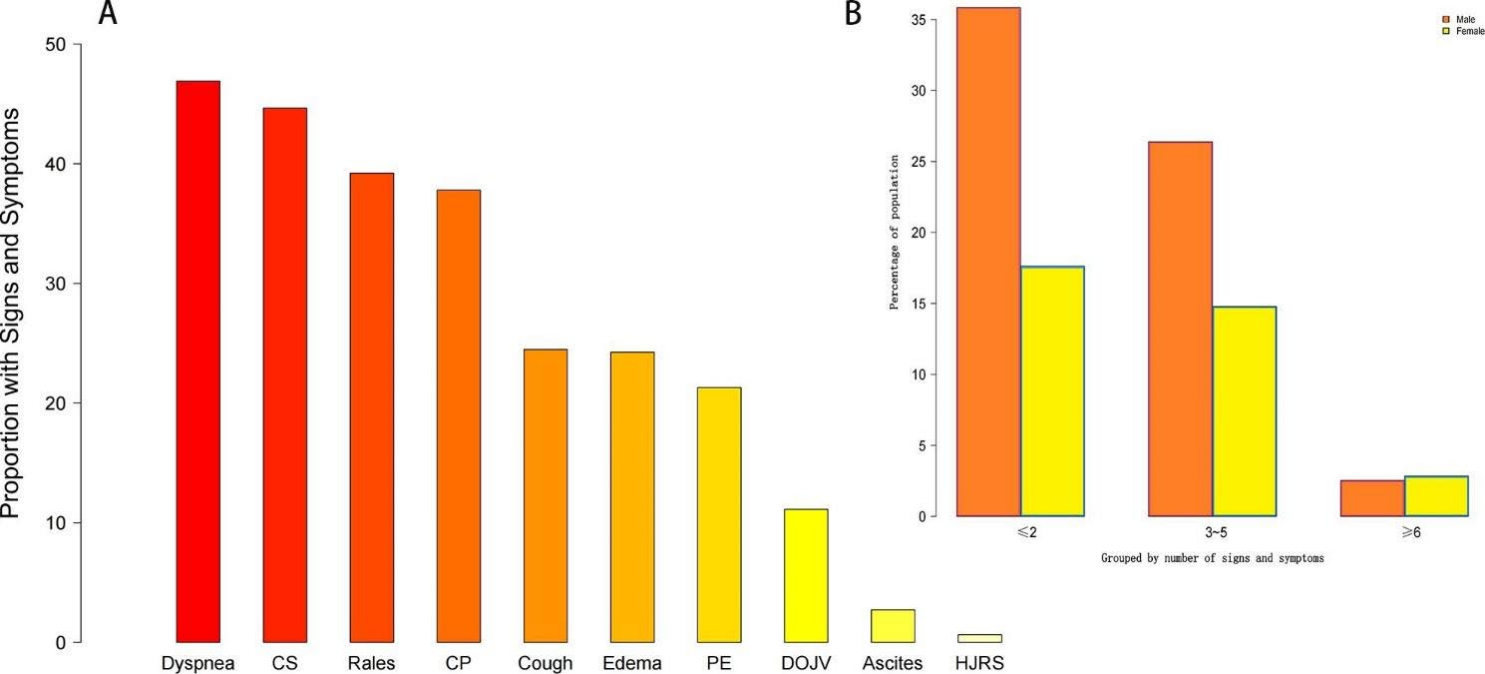
The proportion of each symptom and sign and the proportion after grouping

Compared to group A, patients in group B and group C were older, had higher proportion of females, admission heart rate, systolic blood pressure, New York Heart Association classifications III and IV, atrial fibrillation, diabetes, chronic obstructive pulmonary disease, renal insufficiency, higher level of N-terminal pro-B type natriuretic peptide level, while lower incidence of hyperlipidemia, coronary heart disease and myocardial infarction (Table 1, all p < 0.05). Figure 2 A shows the distribution of each symptom and sign in this cohort and Fig. 2B shows the distribution of the number of symptoms and signs of patients.

**Table 2:** Signs and symptoms as a proportion of clinical outcome

	n(%)	Total Events(%)	Female Events(%)	Male Events(%)
I. All-cause death				
A. Signs and symptoms as a continuous variable (per increment of 1)	1691 (100%)	457(27.03%)	169 (28.36%)	288 (26.30%)
B. Signs and symptoms as a categorical variable				
Group A: ≤2 Signs and symptoms	904(53.46%)	187 (20.69%)	73 (24.50%)	114 (18.81%)
Group B: 3 ~ 5 Signs and symptoms	696(41.16%)	225 (32.33%)*	75 (30.00%)	150 (33.63%)*
Group C: ≥6 Signs and symptoms	91(5.38%)	45 (49.45%)*†	21 (43.75%)*	24 (55.81%)*†
II. Cardiovascular event				
A. Signs and symptoms as a continuous variable (per increment of 1)	1691 (100%)	1160 (68.60%)	412 (69.13%)	748 (68.31%)
B. Signs and symptoms as a categorical variable				
Group A: ≤2 Signs and symptoms	904(53.46%)	568 (62.83%)	193 (64.77%)	375 (61.88%)
Group B: 3 ~ 5 Signs and symptoms	696(41.16%)	513 (73.71%)*	177 (70.80%)	336 (75.34%)*
Group C: ≥6 Signs and symptoms	91(5.38%)	79 (86.81%)*†	42 (87.50%)*	37 (86.05%)*

*P < 0.05 vs. Group A

†P < 0.05 vs. Group B

**Table 3:** Association Between Burden of Signs and Symptoms and Clinical Outcomes

	Female		Male		Total	
	Hazard ratio (95% CI)	*P-value*	Hazard ratio (95% CI)	*P-value*	Hazard ratio (95% CI)	*P-value*
I. All-cause death						
A. Signs and symptoms as a continuous variable (per increment of 1)						
Non-adjusted	1.10 (1.01, 1.19)	**0.0262**	1.24 (1.16, 1.33)	**< 0.0001**	1.18 (1.12, 1.24)	**< 0.0001**
Model I	1.09 (1.01, 1.19)	**0.0323**	1.16 (1.08, 1.25)	**< 0.0001**	1.14 (1.08, 1.20)	**< 0.0001**
Model II	1.05 (0.96, 1.15)	0.2692	1.10 (1.02, 1.19)	**0.0094**	1.08 (1.02, 1.14)	**0.0054**
B. Signs and symptoms as a categorical variable						
Non-adjusted						
Group A: ≤2 Signs and symptoms	Ref.		Ref.		Ref.	
Group B: 3 ~ 5 Signs and symptoms	1.27 (0.92, 1.75)	0.1444	1.96 (1.53, 2.50)	**< 0.0001**	1.68 (1.38, 2.04)	**< 0.0001**
Group C: ≥6 Signs and symptoms	1.94 (1.19, 3.15)	**0.0075**	4.05 (2.61, 6.30)	**< 0.0001**	2.90 (2.09, 4.04)	**< 0.0001**
Model I						
Group A: ≤2 Signs and symptoms	Ref.		Ref.		Ref.	
Group B: 3 ~ 5 Signs and symptoms	1.24 (0.90, 1.71)	0.1891	1.58 (1.23, 2.02)	**0.0003**	1.46 (1.20, 1.78)	**0.0001**
Group C: ≥6 Signs and symptoms	1.90 (1.17, 3.09)	**0.0097**	3.13 (2.01, 4.89)	**< 0.0001**	2.52 (1.81, 3.50)	**< 0.0001**
Model II						
Group A: ≤2 Signs and symptoms	Ref.		Ref.		Ref.	
Group B: 3 ~ 5 Signs and symptoms	1.07 (0.75, 1.51)	0.7086	1.36 (1.05, 1.77)	**0.0195**	1.25 (1.02, 1.54)	**0.0335**
Group C: ≥6 Signs and symptoms	1.36 (0.81, 2.30)	0.2489	2.41 (1.51, 3.85)	**0.0002**	1.89 (1.34, 2.66)	**0.0003**
II. Cardiovascular event						
A.Signs and symptoms as a continuous variable (per increment of 1)						
Non-adjusted	1.11 (1.05, 1.17)	**0.0001**	1.15 (1.10, 1.20)	**< 0.0001**	1.13 (1.10, 1.17)	**< 0.0001**
Model I	1.11 (1.05, 1.17)	**0.0001**	1.13 (1.08, 1.18)	**< 0.0001**	1.12 (1.08, 1.16)	**< 0.0001**
Model II	1.09 (1.03, 1.16)	**0.0032**	1.11 (1.06, 1.17)	**< 0.0001**	1.10 (1.06, 1.14)	**< 0.0001**
B.Signs and symptoms as a categorical variable						
Non-adjusted						
Group A: ≤2 Signs and symptoms	Ref.		Ref.		Ref.	
Group B: 3 ~ 5 Signs and symptoms	1.20 (0.98, 1.47)	0.0821	1.48 (1.28, 1.72)	**< 0.0001**	1.37 (1.22, 1.55)	**< 0.0001**
Group C: ≥6 Signs and symptoms	2.20 (1.57, 3.07)	**< 0.0001**	2.19 (1.56, 3.07)	**< 0.0001**	2.22 (1.75, 2.82)	**< 0.0001**
Model I						
Group A: ≤2 Signs and symptoms	Ref.		Ref.		Ref.	
Group B: 3 ~ 5 Signs and symptoms	1.20 (0.98, 1.47)	0.0810	1.41 (1.21, 1.63)	**< 0.0001**	1.33 (1.18, 1.50)	**< 0.0001**
Group C: ≥6 Signs and symptoms	2.21 (1.58, 3.09)	**< 0.0001**	2.05 (1.46, 2.88)	**< 0.0001**	2.15 (1.69, 2.72)	**< 0.0001**
Model II						
Group A: ≤2 Signs and symptoms	Ref.		Ref.		Ref.	
Group B: 3 ~ 5 Signs and symptoms	1.13 (0.90, 1.41)	0.2824	1.33 (1.14, 1.56)	**0.0003**	1.24 (1.09, 1.41)	**0.0009**
Group C: ≥6 Signs and symptoms	1.96 (1.37, 2.82)	**0.0003**	1.89 (1.33, 2.68)	**0.0004**	1.92 (1.49, 2.45)	**< 0.0001**

Hazard ratios from Cox proportional hazards regressions. Group A: ≤2 Signs and symptoms; Group B: 3 ~ 5 Signs and symptoms; Group C: ≥6 Signs and symptoms.Bold represent significant values (p < 0.05).

Non-adjusted model adjust for: None.

Model I model adjust for: age.

Model II model adjust for: age; systolic blood pressure; heart rate; New York Heart Association functional class; coronary heart disease; hyperlipidemia; atrial fibrillation; diabetes; myocardial infarction; renal insufficiency; hypertension; stroke; current smoker; Current drinker.

Abbreviation: CI = confidence interval

### Clinical outcomes

After a median follow-up of 33 months (20–50 months), all-cause deaths occurred in 457 patients, and CV events occurred in 977 patients.

Comparison on incidence of all-cause mortality and CV events between groups is presented in Table 2. All-cause mortality in Group A was 20.69%, compared to 32.33% in Group B and 49.45% in Group C, revealing significant differences among the three groups (P < 0.05 for all comparisons). This discrepancy persisted in the male patient population; however, a statistically significant difference was only observed among female patients between Group C, and Group A. Cardiovascular events were 62.83% in Group A, 73.71% in Group B, and 86.81% in Group C, with significant differences noted among the three groups (P < 0.05 for all comparisons). Group B and Group C exhibited significant differences in male patients compared to Group A, while no notable difference was observed between Group B and Group C. In female patients, a statistically significant difference was only observed between Group C and Group A.

**Fig. 3:**
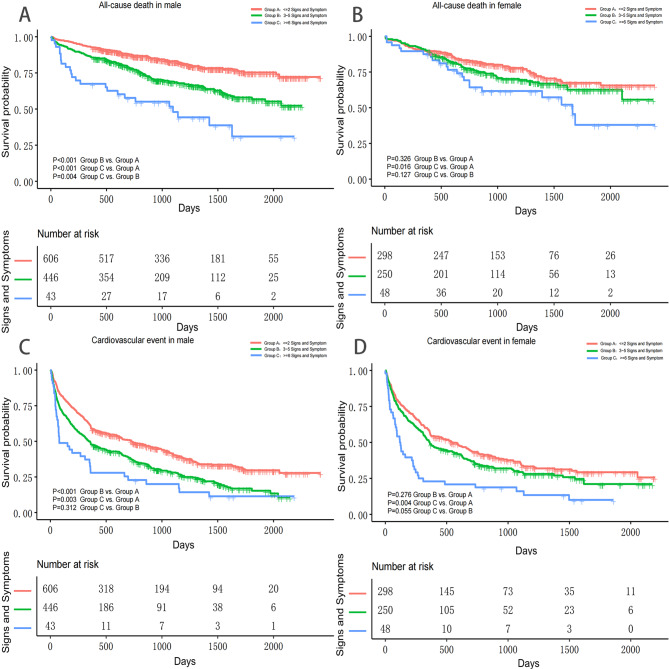
Cumulative Incidence of All-cause death and cardiovascular event. **A**. Cumulative All-cause death in male **B**. Cumulative All-cause death in female **C**. Cumulative Cardiovascular event in male **D**. Cumulative Cardiovascular event in female

Before confounder adjustment, analysis showed that the risk of all-cause mortality increased with each increase on the number of symptoms and signs (HR 1.18; 95% CI 1.12–1.24; P < 0.0001; male HR 1.24, 95% CI 1.16–1.33, P < 0.0001; female HR 1.10; 95% CI 1.01–1.19; P = 0.0262) (Table 3, model IA). Similarly, each increase on the number of symptoms and signs was also associated with an increased risk of CV events (HR 1.13; 95% CI 1.10–1.17; P < 0.0001) (male HR 1.15, 95% CI 1.10–1.20, P < 0.0001; female HR 1.11; 95% CI 1.05–1.17; P = 0.0001) (Table 3, model IIA). When symptoms and signs were analyzed as categorical variables, in male patients, risk of all-cause mortality was significantly higher in group B (HR 1.96; 95% CI 1.53–2.50; P < 0.0001) and group C (HR 4.05; 95% CI 2.61–6.30; P < 0.0001) than in group A (Fig. 3A; Table 3, model IB). In female patients, risk of all-cause mortality was significantly higher in group C than in group A (HR 1.94; 95% CI 1.19–3.15; P = 0.0075), which was similar between group B and group A (HR 1.27; 95% CI 0.92–1.75; P = 0.1444) (Fig. 3B; Table 3, model IB). In male patients, risk of CV events was significantly higher in group B (HR 1.48; 95% CI 1.28–1.72; P < 0.0001) and group C (HR 2.19; 95% CI 1.56–3.07; P < 0.0001) than in group A (Fig. 3C; Table 3, model IIB). In female patients, risk of CV events was significantly higher in group C than in group A (HR 2.20; 95% CI 1.57–3.07; P < 0.0001), which was similar between group B and group A (HR 1.20; 95% CI 0.98–1.47; P = 0.0821) (Fig. 3D; Table 3, model IIB).

After adjusting for age (model I), each increase in the number of symptoms and signs was associated with an increased risk of all-cause mortality (HR 1.14; 95% CI 1.08–1.20; P < 0.0001) and CV events (HR 1.12; 95% CI 1.08–1.16; P < 0.0001) in both sexes. When symptoms and signs were treated as categorical variables, compared to group A, male patients in groups B (HR 1.58; 95% CI 1.23–2.02; P = 0.0003) and C (HR 3.13; 95% CI 2.01–4.89; P < 0.0001) faced an increased risk of all-cause mortality. In female patients, the risk of all-cause mortality was higher in group C than in group A (HR 1.90; 95% CI 1.17–3.09; P = 0.0097), but there was no significant difference in all-cause mortality between group B and group A (HR 1.24; 95% CI 0.90–1.71; P = 0.1891). Compared to group A, male patients in groups B (HR 1.41; 95% CI 1.21–1.63; P < 0.0001) and C (HR 2.05; 95% CI 1.46–2.88; P < 0.0001) were associated with an increased risk of CV events. In female patients, the risk of CV events was higher in group C than in group A (HR 2.21; 95% CI 1.58–3.09; P < 0.0001), but there was no significant difference in CV events between group B and group A (HR 1.20; 95% CI 0.98–1.47; P = 0.0810) (Table 3).

**Fig. 4:**
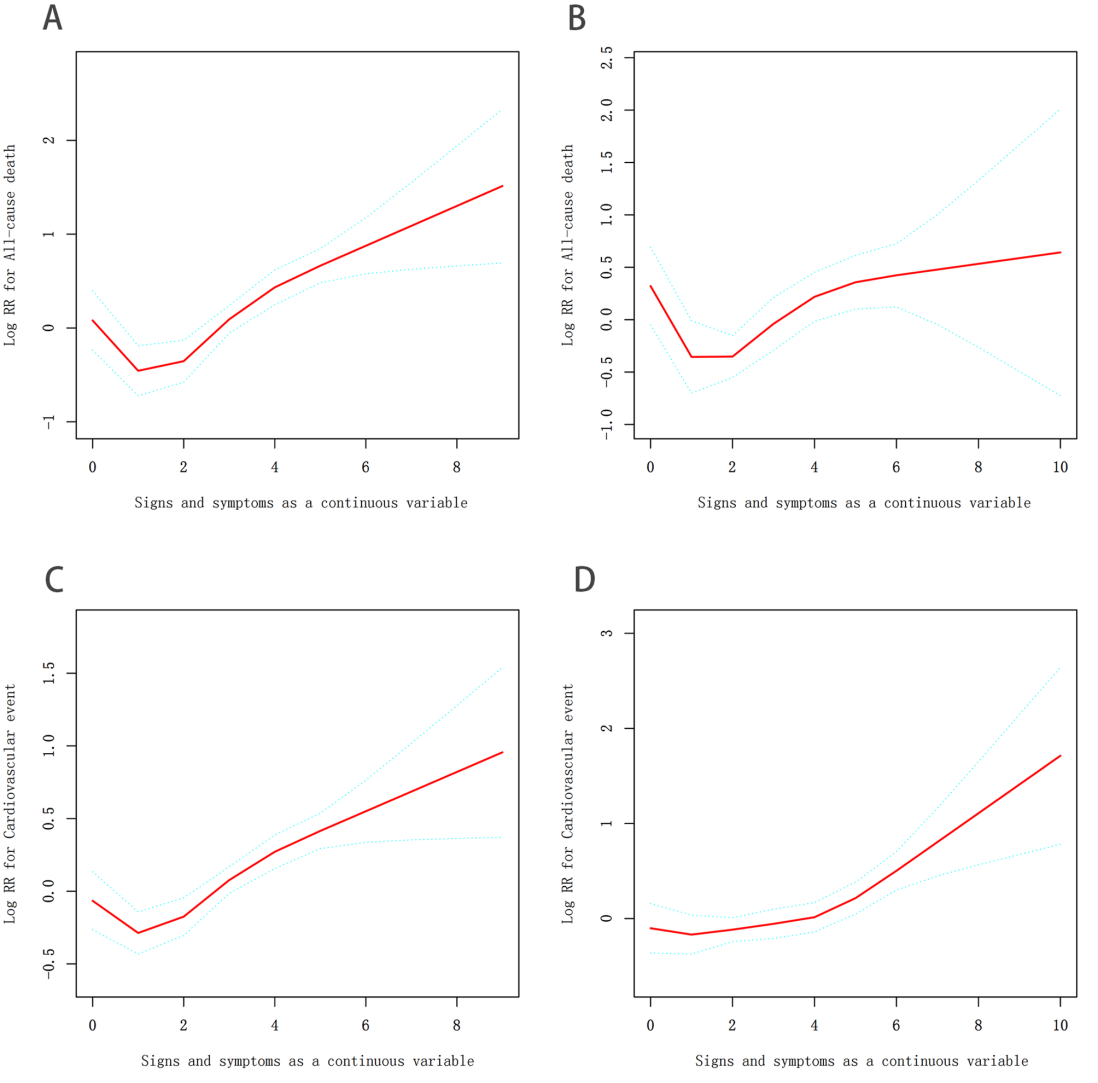
Curve fitting between Signs and symptoms as a continuous variable and outcome events. **A**. Curve fitting of signs and symptoms to All-cause death in male. **B**. Curve fitting of signs and symptoms to All-cause death in female. **C**. Curve fitting of signs and symptoms to Cardiovascular event in male. **D**. Curve fitting of signs and symptoms to Cardiovascular event in female. Curve fitting adjust for: age; systolic blood pressure; heart rate; New York Heart Association functional class; coronary heart disease; hyperlipidemia; atrial fibrillation; diabetes; myocardial infarction; renal insufficiency; hypertension; stroke; current smoker; Current drinker

**Table 4:** Cox Proportional Hazards Regression Model Analysis for Risk of Outcomes

	Univariable			Multivariable	
Variable	Hazard ratio (95% CI)	Wald	*P-value*	Hazard ratio (95% CI)	*P-value*
I. All-cause death					
A.Signs and symptoms as a continuous variable (per increment of 1)					
Age per year	1.049 (1.040, 1.059)	**113.4**	**< 0.001**	1.048 (1.038, 1.057)	**< 0.001**
Renal insufficiency	2.018 (1.665, 2.446)	**51.12**	**< 0.001**	1.776 (1.452, 2.173)	**< 0.001**
Signs and symptoms	1.177 (1.118, 1.239)	**38.89**	**< 0.001**	1.082 (1.024, 1.143)	**0.005**
Myocardial infarction	0.577 (0.478, 0.696)	**33.14**	**< 0.001**	0.624 (0.503, 0.775)	**< 0.001**
Stroke	1.748 (1.381, 2.213)	**21.56**	**< 0.001**	1.493 (1.174, 1.898)	**0.001**
NYHA class IV/III vs. II	1.576 (1.297, 1.916)	**20.84**	**< 0.001**	1.141 (0.930, 1.400)	0.207
Atrial fibrillation	1.507 (1.212, 1.872)	**13.66**	**< 0.001**	1.123 (0.889, 1.420)	0.331
Hypertension	1.430 (1.157, 1.766)	**10.99**	**< 0.001**	1.055 (0.842, 1.322)	0.642
Hyperlipidemia	0.707 (0.552, 0.906)	**7.53**	**0.006**	0.865 (0.669, 1.118)	0.268
Heart rate	1.005 (1.001, 1.010)	**6.59**	**0.010**	1.005 (1.000, 1.009)	**0.043**
Diabetes	1.267 (1.048, 1.533)	**5.96**	**0.015**	1.247 (1.021, 1.524)	**0.031**
Systolic blood pressure	1.003 (1.000, 1.007)	3.72	0.054		
Current drinker	0.763 (0.538, 1.082)	2.3	0.129		
Current smoker	0.859 (0.703, 1.050)	2.2	0.138		
Coronary heart disease	0.861 (0.695, 1.066)	1.88	0.170		
Male vs. Female	0.907 (0.750, 1.097)	1.01	0.316		
B.Signs and symptoms as a categorical variable(All other variables remain the same)					
≤ 2 Signs and symptoms	Ref.				
3 ~ 5 Signs and symptoms	1.682 (1.385, 2.042)		**< 0.001**	1.317 (1.070, 1.621)	**0.009**
≥6 Signs and symptoms	2.920 (2.109, 4.044)		**< 0.001**	1.982 (1.402, 2.801)	**< 0.001**
II. Cardiovascular event					
A.Signs and symptoms as a continuous variable (per increment of 1)					
Signs and symptoms	1.135 (1.097, 1.174)	**53.68**	**< 0.001**	1.099 (1.060, 1.140)	**< 0.001**
Age per year	1.015 (1.010, 1.020)	**33.62**	**< 0.001**	1.012 (1.007, 1.017)	**< 0.001**
Renal insufficiency	1.372 (1.204, 1.563)	**22.58**	**< 0.001**	1.248 (1.088, 1.431)	**0.002**
Atrial fibrillation	1.356 (1.173, 1.568)	**16.95**	**< 0.001**	1.251 (1.069, 1.464)	**0.005**
Hypertension	1.246 (1.098, 1.414)	**11.61**	**< 0.001**	1.094 (0.954, 1.255)	0.197
Diabetes	1.211 (1.073, 1.366)	**9.58**	**0.002**	1.162 (1.024, 1.319)	**0.020**
Myocardial infarction	0.842 (0.750, 0.944)	**8.6**	**0.003**	0.888 (0.773, 1.021)	0.095
Heart rate	1.004 (1.001, 1.007)	**7.7**	**0.006**	1.001 (0.998, 1.004)	0.423
NYHA class IV/III vs. II	1.176 (1.046, 1.322)	**7.39**	**0.007**	0.991 (0.876, 1.121)	0.882
Current drinker	0.752 (0.607, 0.930)	**6.89**	**0.009**	0.816 (0.645, 1.031)	0.088
Current smoker	0.859 (0.759, 0.973)	**5.72**	**0.017**	0.939 (0.810, 1.088)	0.403
Systolic blood pressure	1.003 (1.000, 1.005)	**5.65**	**0.017**	1.001 (0.999, 1.003)	0.439
Stroke	1.168 (0.986, 1.383)	3.22	0.073		
Hyperlipidemia	0.898 (0.777, 1.038)	2.11	0.146		
Male vs. Female	0.933 (0.828, 1.053)	1.26	0.262		
Coronary heart disease	0.980 (0.853, 1.125)	0.09	0.770		
B. Signs and symptoms as a categorical variable(All other variables remain the same)					
≤ 2 Signs and symptoms	Ref.				
3 ~ 5 Signs and symptoms	1.377 (1.221, 1.551)		**< 0.001**	1.271 (1.119, 1.443)	**< 0.001**
≥6 Signs and symptoms	2.236 (1.766, 2.831)		**< 0.001**	1.955 (1.524, 2.508)	**< 0.001**

Bold represent significant values (p < 0.05).

Abbreviations: CI = confidence interval; NYHA = New York Heart Association classification.

After adjusting for all covariates (model II), the risk of all-cause mortality increased with the increasing number of symptoms and signs in male patients (HR 1.10; 95% CI 1.02–1.19; P = 0.0094), while the risk increase was not significant in female patients (HR 1.05; 95% CI 0.96–1.15; P = 0.2692) (Fig. 4A, B; Table 3). Each increase on number of symptoms and signs was associated with an increased risk of cardiovascular (CV) events (HR 1.10; 95% CI 1.06–1.14; P < 0.0001) in both male patients (HR 1.11, 95% CI 1.06–1.17, P < 0.0001) and female patients (HR 1.09; 95% CI 1.03–1.16; P = 0.0032) (Fig. 4C, D; Table 3). When symptoms and signs were treated as categorical variables, compared with group A, the increase of symptoms and signs was still associated with increased risk of all-cause mortality in group B (HR 1.36; 95% CI 1.05–1.77; P = 0.0195) and in group C (HR 2.41; 95% CI 1.51–3.85; P = 0.0002) in male patients (Table 3, Model IB). In female patients, the differences in all-cause mortality between group A and group B (HR 1.07; 95% CI 0.75–1.51; P = 0.7086) and between group A and group C (HR 1.36; 95% CI 0.81–2.30; P = 0.2489) were not significant (Table 3). Compared with group A, male patients in group B (HR 1.33; 95% CI 1.14–1.56; P = 0.0003) and group C (HR 1.89; 95% CI 1.33–2.68; P = 0.0004) faced increased risk of CV events (Table 3). In female patients, the risk of CV events was higher in group C than that in group A (HR 1.96; 95% CI 1.37–2.82; P = 0.0003), but there was no significant difference between group B and group A (HR 1.13; 95% CI 0.90–1.41; P = 0.2824) (Table 3).

**Fig. 5:**
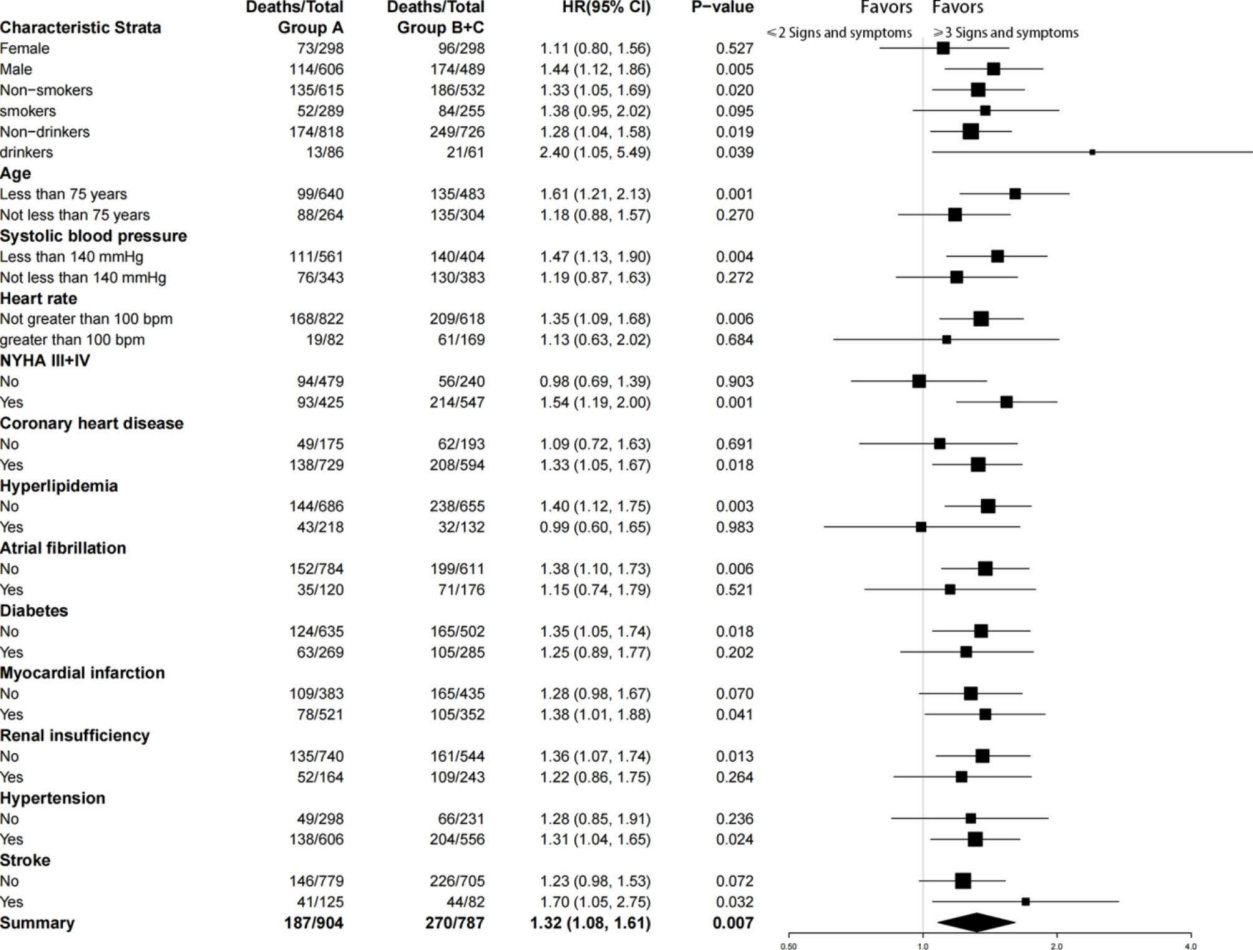
Multivariable Stratified Analyses of the Association Between Signs and symptoms as a categorical variable and All-cause death. Multivariable model adjust for: age; sex; systolic blood pressure; heart rate; New York Heart Association functional class; coronary heart disease; hyperlipidemia; atrial fibrillation; diabetes; myocardial infarction; renal insufficiency; hypertension; stroke; current smoker; Current drinker

**Table 5:** Association Between Individual Signs and Symptoms and Clinical Outcomes

	Female		Male		Total	
	Adjusted Hazard ratio(95% CI)*	P-value	Adjusted Hazard ratio(95% CI)*	P-value	Adjusted Hazard ratio(95% CI)*	P-value
All-cause death						
Chest pain	0.71 (0.47, 1.09)	0.1139	0.55 (0.40, 0.75)	**0.0001**	0.61 (0.47, 0.78)	**< 0.0001**
Chest suffocation	0.79 (0.58, 1.09)	0.1479	1.06 (0.84, 1.34)	0.6295	0.97 (0.80, 1.17)	0.7259
Dyspnea	1.04 (0.74, 1.46)	0.8256	1.53 (1.18, 1.97)	**0.0013**	1.31 (1.07, 1.60)	**0.0096**
Cough	1.22 (0.86, 1.73)	0.2627	1.33 (1.02, 1.73)	**0.0339**	1.29 (1.05, 1.59)	**0.0160**
Jugular venous distension	1.33 (0.88, 1.99)	0.1735	1.06 (0.72, 1.56)	0.7710	1.18 (0.90, 1.56)	0.2352
Hepatojugular reflux sign	1.97 (0.57, 6.74)	0.2815	4.30 (1.56, 11.83)	**0.0047**	2.72 (1.26, 5.88)	**0.0108**
Rales	1.30 (0.94, 1.80)	0.1083	1.23 (0.96, 1.57)	0.1077	1.25 (1.03, 1.52)	**0.0260**
Edema	1.28 (0.91, 1.80)	0.1615	1.18 (0.91, 1.54)	0.2198	1.24 (1.00, 1.52)	**0.0454**
Ascites	1.05 (0.50, 2.19)	0.9045	2.01 (0.96, 4.17)	0.0622	1.44 (0.86, 2.40)	0.1646
Pleural effusion	1.32 (0.94, 1.87)	0.1113	1.51 (1.15, 1.96)	**0.0025**	1.42 (1.15, 1.75)	**0.0010**
Cardiovascular event						
Chest pain	0.92 (0.72, 1.18)	0.5164	0.74 (0.61, 0.88)	**0.0007**	0.81 (0.70, 0.93)	**0.0036**
Chest suffocation	1.03 (0.85, 1.26)	0.7507	1.15 (0.99, 1.33)	0.0597	1.10 (0.98, 1.24)	0.1096
Dyspnea	1.30 (1.05, 1.61)	**0.0154**	1.37 (1.17, 1.60)	**< 0.0001**	1.31 (1.16, 1.49)	**< 0.0001**
Cough	1.27 (1.00, 1.60)	**0.0459**	1.27 (1.07, 1.51)	**0.0068**	1.25 (1.09, 1.44)	**0.0015**
Jugular venous distension	1.37 (1.04, 1.81)	**0.0264**	1.22 (0.96, 1.55)	0.1039	1.28 (1.07, 1.53)	**0.0069**
Hepatojugular reflux sign	0.95 (0.30, 3.03)	0.9277	0.96 (0.35, 2.59)	0.9341	0.99 (0.47, 2.09)	0.9784
Rales	1.26 (1.02, 1.55)	**0.0300**	1.18 (1.01, 1.38)	**0.0325**	1.20 (1.06, 1.36)	**0.0037**
Edema	1.46 (1.16, 1.84)	**0.0013**	1.35 (1.13, 1.61)	**0.0009**	1.36 (1.19, 1.57)	**< 0.0001**
Ascites	1.08 (0.66, 1.76)	0.7654	1.73 (1.03, 2.92)	**0.0400**	1.21 (0.85, 1.72)	0.2940
Pleural effusion	0.98 (0.78, 1.24)	0.8794	1.37 (1.14, 1.64)	**0.0007**	1.20 (1.04, 1.39)	**0.0105**

Hazard ratios from Cox proportional hazards regressions. Bold represent significant values (p < 0.05).

*Adjusted for age; systolic blood pressure; heart rate; New York Heart Association functional class; coronary heart disease; hyperlipidemia; atrial fibrillation; diabetes; myocardial infarction; renal insufficiency; hypertension; stroke; current smoker; Current drinker.

Abbreviations:CI = confidence interval.

A. Cumulative All-cause death in male

B. Cumulative All-cause death in female

C. Cumulative Cardiovascular event in male

D. Cumulative Cardiovascular event in female

A. Curve fitting of signs and symptoms to All-cause death in male

B. Curve fitting of signs and symptoms to All-cause death in female

C. Curve fitting of signs and symptoms to Cardiovascular event in male

D. Curve fitting of signs and symptoms to Cardiovascular event in female

Curve fitting adjust for: age; systolic blood pressure; heart rate; New York Heart Association functional class; coronary heart disease; hyperlipidemia; atrial fibrillation; diabetes; myocardial infarction; renal insufficiency; hypertension; stroke; current smoker; Current drinker

Multivariable model adjust for: age; sex; systolic blood pressure; heart rate; New York Heart Association functional class; coronary heart disease; hyperlipidemia; atrial fibrillation; diabetes; myocardial infarction; renal insufficiency; hypertension; stroke; current smoker; Current drinker

Adjusted HR: 1.10; 95% CI 1.02–1.19; P = 0.0094 for male Adjusted HR 1.11, 95% CI 1.06–1.17, P < 0.0001 for male

Adjusted HR 1.05; 95% CI 0.96–1.15; P = 0.2692 for female Adjusted HR 1.09; 95% CI 1.03–1.16; P = 0.0032 for female

### Independent risk factors of outcomes

Multivariate Cox regression models were used to identify risk factors independently associated with outcome events (Table 4). Parameters with a significance level of P < 0.05 from univariate Cox regression analysis were included in the multivariate model. Results showed that following factors were independently associated with all-cause death, including older age, renal insufficiency, higher number of signs and symptoms (≥ 3, hazard ratio [HR] 1.317, 95% confidence interval [CI] 1.070–1.621, P = 0.009; ≥6, HR 1.982, 95% CI 1.402–2.801, P < 0.001), myocardial infarction, stroke, faster heart rate on admission, and diabetes (all P < 0.05). Similarly, higher number of signs and symptoms (≥ 3, HR 1.271, 95% CI 1.119–1.443, P < 0.001; ≥6, HR 1.955, 95% CI 1.524–2.508, P < 0.001), older age, renal insufficiency, atrial fibrillation, and diabetes were independently associated with cardiovascular events (all P < 0.05).

**Fig. 6:**
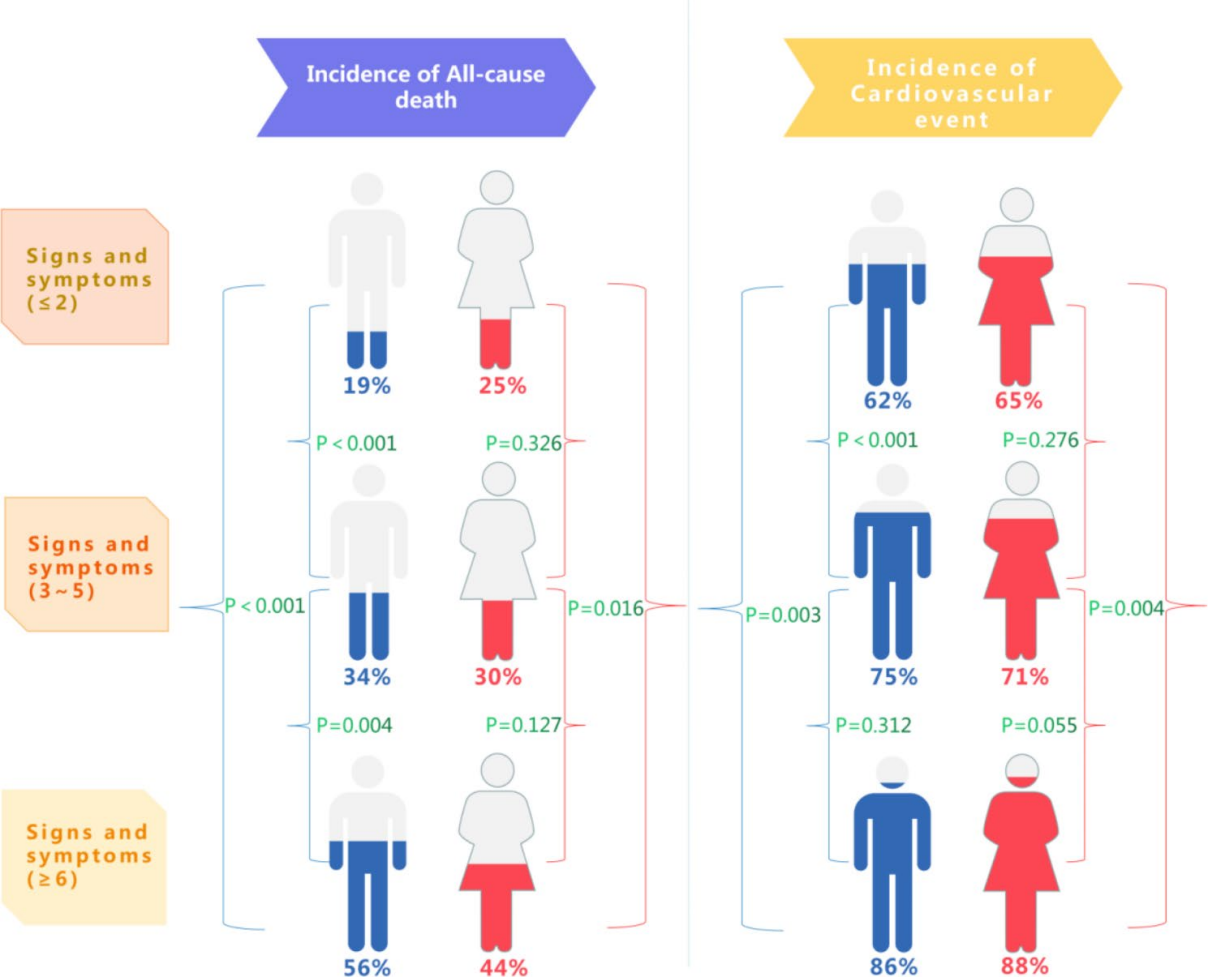
Central illustration: Core results. Adjusted HR: 1.10; 95% CI 1.02-1.19; P=0.0094 for male. Adjusted HR 1.11, 95% CI 1.06-1.17, P<0.0001 for male. Adjusted HR 1.05; 95% CI 0.96-1.15; P=0.2692 for female, Adjusted HR 1.09; 95% CI 1.03-1.16; P=0.0032 for female

In patients with ≥ 3 symptoms or signs, the following characteristics were associated with increased risk of all-cause mortality: male sex, age ≥ 75 years, systolic blood pressure ≥ 140 mmHg, heart rate < 100 beats/min, NYHA III + IV, presence of coronary heart disease, myocardial infarction, hypertension, stroke, even in the absence of hyperlipidemia, atrial fibrillation, diabetes, and renal insufficiency (Fig. 5).

### Impact of individual signs and symptoms on outcome events

We investigated the relationship between individual signs and symptoms and clinical outcomes in our patients. Results showed that chest pain, dyspnea, cough, hepatojugular reflux sign, and pleural effusion were independently associated with all-cause mortality in male patients. Dyspnea, cough, rales, and edema were independently associated with cardiovascular events (CV events) in all patients regardless of sex. Chest pain, ascites, and pleural effusion were independently associated with CV events in male patients, while jugular venous distension was independently associated with CV events in female patient (all P < 0.05) (Table 5).

Additionally, we observed the impact of initial LVEF on the outcome of our patients. The patients were divided into constant mrEF (mEF) group (n = 1168), patients from rEF to mrEF (rEF) group (n = 125) and from pEF to mrEF (pEF) group (n = 398). As shown in Table S2, compared to constant mrEF group, risk for all-cause death and cardiovascular events was significantly higher in rEF and pEF groups as compared to mrEF group. Table S3 illustrates the association between symptom burden and clinical outcomes in these groups. We used both continuous and categorical analysis to establish the hazard ratio of symptoms. The results showed a trend of increasing hazard ratio with an increasing number of symptoms for both all-cause death and cardiovascular events across all groups.

## Discussion

The main finding of present study are as follows: In HFmrEF patients, the risk of all-cause mortality and CV events increased with increasing symptoms and signs (Fig. 6 central illustration).

Our results thus indicate the prognostic importance of careful inquiry on HF symptoms and related physical examination in HFmrEF patients. To our best knowledge, this is the first clinical report highlighting the importance of careful inquiring of HF symptoms and signs on risk stratification in HFmrEF patients.

Our finding in HFmrEF cohort is similar as that of HFpEF patients. A total of 4725 patients with HFpEF were included in the post-hoc analysis of PARAGONHF [9], it was shown that high HF sign and symptom burden was associated with an increased risk of heart failure admission and cardiovascular death [9]. Our results added the prognostic value of symptoms and signs on all-cause death in HFmrEF patients. Thus, a detailed history and physical examination might provide helpful information for risk stratification on cardiovascular outcome in both HFpEF and HFmrEF patients. Patients with HFpEF have the same typical heart failure symptoms as those with HFrEF [16]. Previous studies examining various combinations of signs and symptoms in inpatients with HFrEF showed a similarly increased cardiovascular risk as reported in the post-hoc analysis of PARAGONHF [10–12]. Taken together, careful assessment of signs and inquiry of HF symptoms are valuable for the risk stratification of HF patients with various EF categories. This economical tool should be applied more frequently in daily clinical practice to treat the patients in a more cost-effective fashion.

According to current HF guideline, treatment should focus on preventing progressive adverse cardiac remodeling and reducing mortality and hospitalization rates [5, 17]. Even with the modern HF treatment options, the patient’s symptom burden still increases over time [18] and serves as an important index related to worsening quality of life [17]. It is to note that some HF patients may also be less aware of their real severity on symptoms [19] and even the milder symptoms of CHF can cause problems in patients’ lives [20]. Thus, physicians should pay more attention to obtain real information on symptoms in HF patients. The central task of hospitalization of CHF patients is symptom control [21], and in fact, prioritizing symptom management in the clinic can reduce re-hospitalization and the cost of treatment [1]. Thus, symptom control should be highlighted in hospitalized HF patients with various EF categories.

### Study limitations

This study has several limitations. First, an inherent limitation of this retrospective study design is the inability to comprehensively capture and verify all potentially significant signs and symptoms of heart failure such as orthopnea, bendopnea, and Paroxysmal Nocturnal Dyspnea (PND). These symptoms were not systematically documented in the medical records used for this study. As a result, their absence may potentially impact the robustness of our findings. Future prospective studies are needed to incorporate these important symptoms for a more comprehensive assessment of heart failure. Second, our study recruited patients from a single heart center in China and, future studies including patients from elsewhere are needed to demonstrate the consistency of the findings. Third, because the limited use of angiotensin receptor neprilysin inhibitors and sodium-dependent glucose transporters, two inhibitors, which has only recently been introduced in our hospital, might affect the outcome of patients in our cohort. Fourth, present study did not explore the prognostic value of signs and symptoms in HFmrEF patients on patients with different severity of biomarkers derived from imaging and laboratory tests, and future studies are needed to explore patients with similar signs and symptoms but varied severity of biomarkers derived from imaging and laboratory tests.

### Clinical implication

The study highlights the value of physical examination skills in HF. A better understanding of the prognostic impact of signs and symptoms in patients with HFmrEF may help develop strategies to improve the outcome of HFmrEF patients, future studies are needed to observe if more intensive HF care and management might improve the outcome of HFmrEF patients with high symtoms and signs burden at admission. Clinicians should not only rely on imaging and laboratory tests, but also aware the prognostic importance of refining signs and symptoms. A detailed physical examination is critical in determining the patient’s condition when imaging and laboratory test results are unavailable to the first consulting physician.

## Conclusions

Despite the increasing availability of imaging and laboratory tests for diagnosing heart failure, a detailed physical examination remains highly significant in clinical practice. In HFmrEF patients, the risk of all-cause mortality increased with increasing symptoms and signs. Higher number of symptoms and signs is also associated with increased risk of CV events in HFmrEF patients. Future prospective studies are warranted to know if intensive HF management strategy focusing on HF symptoms and signs control could improve the outcome of HFmrEF patients with high symptoms and signs burden.

### Electronic supplementary material

Below is the link to the electronic supplementary material.

Additional File 1: Baseline features of Serology and Echocardiography are stratified by the burden of signs and symptoms and then by gender.

## Data Availability

The datasets generated and analyzed during the current study are not publicly available due the database owner is reluctant to make them public but are available from the corresponding author upon reasonable request.
