# Supplementary material for: Impact of signs and symptoms on the prognosis of patients with HFmrEF
Source: BMC Cardiovasc Disord. 2023 Aug 24;23:420. doi: 10.1186/s12872-023-03436-z (PMC10464266; doi:10.1186/s12872-023-03436-z)
Supplement: Supplementary file 1 — Additional File 1: Baseline features of Serology and Echocardiography are stratified by the burden of signs and symptoms and then by gender. [file 12872_2023_3436_MOESM1_ESM.docx]

| **Table S1** Baseline features of Serology and Echocardiography are stratified by the burden of signs and symptoms and then by gender. | | | |
| --- | --- | --- | --- |
|  | Signs and Symptoms | | |
|  | Group A：≤2 | Group B：3~5 | Group C：≥6 |
| NT-proBNP, pg/ml |  |  |  |
| Male | 3737.6 ± 6655.8 | 7886.3 ± 10058.5* | 13424.6 ± 10844.2*† |
| Female | 5865.1 ± 8245.8‡ | 10215.9 ± 11749.3*‡ | 15490.1 ± 12702.0*†‡ |
| Hemoglobin, g/dL |  |  |  |
| Male | 130.8 ± 21.7 | 123.2 ± 24.2* | 113.5 ± 23.9*† |
| Female | 116.9 ± 18.8‡ | 111.3 ± 20.2*‡ | 104.1 ± 22.1*†‡ |
| Sodium, mmol/L |  |  |  |
| Male | 139.3 ± 3.5 | 139.4 ± 3.7 | 139.7 ± 3.6 |
| Female | 139.1 ± 3.5 | 139.4 ± 3.7 | 139.9 ± 3.9 |
| Uric Acid,µmol/L |  |  |  |
| Male | 359.2 ± 108.6 | 392.3 ± 119.9* | 403.6 ± 157.5* |
| Female | 317.0 ± 109.4‡ | 364.4 ± 117.7*‡ | 409.1 ± 116.7*† |
| eGFR, ml/min/1.73 m2 |  |  |  |
| Male | 78.4 ± 35.1 | 65.5 ± 31.9* | 55.0 ± 32.9*† |
| Female | 70.3 ± 31.0‡ | 61.6 ± 41.0* | 44.4 ± 33.6*†‡ |
| Low density lipoprotein,mmol/L |  |  |  |
| Male | 2.5 ± 1.0 | 2.4 ± 0.9 | 2.4 ± 1.1 |
| Female | 2.6 ± 1.0 | 2.4 ± 1.0 | 2.4 ± 1.1 |
| LVEF , % |  |  |  |
| Male | 44.6 ± 2.8 | 44.2 ± 2.7 | 44.8 ± 2.6 |
| Female | 44.4 ± 2.8 | 44.3 ± 2.7 | 43.9 ± 2.7 |
| LAs (mm) |  |  |  |
| Male | 38.0 ± 5.5 | 40.7 ± 6.5* | 41.8 ± 5.6* |
| Female | 37.8 ± 6.2 | 40.7 ± 6.4* | 41.4 ± 5.0* |
| LVd (mm) |  |  |  |
| Male | 53.8 ± 6.2 | 56.0 ± 7.5* | 56.2 ± 5.7* |
| Female | 51.6 ± 6.2‡ | 53.4 ± 7.8*‡ | 53.8 ± 5.0*‡ |
| RAs (mm) |  |  |  |
| Male | 37.0 ± 5.2 | 39.2 ± 6.9* | 40.8 ± 9.7* |
| Female | 35.7 ± 5.4‡ | 37.6 ± 6.2*‡ | 38.8 ± 7.6*‡ |
| RVd (mm) |  |  |  |
| Male | 20.9 ± 5.3 | 21.6 ± 5.5 | 21.1 ± 5.7 |
| Female | 20.1 ± 4.9‡ | 20.5 ± 4.9‡ | 20.7 ± 5.6 |
| E/e′ |  |  |  |
| Male | 14.1 ± 6.6 | 16.0 ± 7.5* | 19.8 ± 9.3*† |
| Female | 15.7 ± 7.9‡ | 18.2 ± 8.9*‡ | 19.4 ± 7.7* |
| PASP(mmHg) |  |  |  |
| Male | 28.1 ± 14.5 | 35.5 ± 21.5* | 39.1 ± 16.6*† |
| Female | 31.9 ± 15.7‡ | 37.2 ± 15.7* | 43.0 ± 14.9*†‡ |
| The population was classified according by Burden of Signs and Symptoms,and stratified by gender. Values for continuous variables are given as means ±SD.Group A：≤2 Signs and symptoms；Group B：3~5 Signs and symptoms；Group C：≥6 Signs and symptoms.  *Differences from Group A. †Differences from Group B. ‡Differences between men and women.  Values ＜0.05 are generally considered meaningful.  Abbreviations: LVEF:left ventricular ejection fraction;NT-proBNP :N-terminal pro-B type natriureti peptide;  eGFR:estimated glomerular filtration rate;LAs:Left atrial size;LVd: left ventricle dimension; RAs :right atrial size; RVd : right ventricle dimension;E/e′:ratio of early transmitral flow velocity to early mitral annular velocity;  PASP:pulmonary artery systolic pressure. | | | |

| **TABLE S2 Prognostic comparison among groups following stratification based on initial LVEF values** | | | | |
| --- | --- | --- | --- | --- |
|  | All-cause death | | Cardiovascular event | |
| Initial LVEF Group | Hazard ratio (95% CI) | *P-value* | Hazard ratio (95% CI) | *P-value* |
| Group rEF vs Group mrEF | 1.82 (1.33, 2.49) | **0.0002** | 1.41 (1.14, 1.74) | **0.0016** |
| Group pEF vs Group mrEF | 2.19 (1.78, 2.68) | **<0.0001** | 1.44 (1.26, 1.65) | **<0.0001** |
| Group pEF vs Group rEF | 1.20 (0.86, 1.68) | 0.2756 | 1.03 (0.81, 1.29) | 0.8261 |
| Hazard ratios from Cox proportional hazards regressions.Bold represent significant values (p < 0.05).Group mrEF: Initial diagnosis of HFmrEF or Maintain in HFmrEF; Group rEF: HFrEF improved to HFmrEF; Group pEF: HFpEF deteriorated to HFmrEF.  Abbreviation: LVEF=Left Ventricular Ejection Fraction; CI=conﬁdence interval; HFmrEF=Heart failure with mildly reduced ejection fraction; HFrEF=Heart failure with reduced ejection fraction; HFpEF=Heart failure with preserved ejection fraction. | | | | |

| **TABLE S3 The association between symptom burden and clinical outcomes following grouping based on initial LVEF values** | | | | | | |
| --- | --- | --- | --- | --- | --- | --- |
|  | Group mrEF (n=1168) | | Group rEF (n=125) | | Group pEF (n=398) | |
|  | Hazard ratio (95% CI) | *P-value* | Hazard ratio (95% CI) | *P-value* | Hazard ratio (95% CI) | *P-value* |
| **All-cause death:** | | | | | | |
| Signs and symptoms as a continuous variable (per increment of 1) | 1.18 (1.10, 1.27) | **<0.0001** | 1.22 (1.04, 1.44) | **0.0171** | 1.10 (1.01, 1.20) | **0.0238** |
| Signs and symptoms as a categorical variable: | | | | | | |
| Group A:≤2 Signs and symptoms | Ref. |  | Ref. |  | Ref. |  |
| Group B:3~5 Signs and symptoms | 1.59 (1.23, 2.05) | **0.0004** | 2.03 (1.07, 3.87) | **0.0306** | 1.41 (1.00, 2.00) | 0.0507 |
| Group C:≥6 Signs and symptoms | 3.07 (2.02, 4.66) | **<0.0001** | 2.45 (0.80, 7.48) | 0.1151 | 2.32 (1.29, 4.18) | **0.005** |
| **Cardiovascular event:** | | | | | | |
| Signs and symptoms as a continuous variable (per increment of 1) | 1.11 (1.07, 1.16) | **<0.0001** | 1.31 (1.15, 1.49) | **<0.0001** | 1.13 (1.06, 1.20) | **<0.0001** |
| Signs and symptoms as a categorical variable: | | | | | | |
| Group A:≤2 Signs and symptoms | Ref. |  | Ref. |  | Ref. |  |
| Group B:3~5 Signs and symptoms | 1.31 (1.13, 1.52) | **0.0003** | 1.56 (1.03, 2.37) | **0.0374** | 1.37 (1.08, 1.74) | **0.0103** |
| Group C:≥6 Signs and symptoms | 2.07 (1.54, 2.76) | **<0.0001** | 6.25 (2.56, 15.29) | **<0.0001** | 2.24 (1.42, 3.54) | **0.0005** |
| Hazard ratios from Cox proportional hazards regressions.Bold represent significant values (p < 0.05).Group mrEF: Initial diagnosis of HFmrEF or Maintain in HFmrEF; Group rEF: HFrEF improved to HFmrEF; Group pEF: HFpEF deteriorated to HFmrEF. Group A:≤2 Signs and symptoms; Group B:3~5 Signs and symptoms; Group C:≥6 Signs and symptoms.  Abbreviation: LVEF=Left Ventricular Ejection Fraction; CI=conﬁdence interval; HFmrEF=Heart failure with mildly reduced ejection fraction; HFrEF=Heart failure with reduced ejection fraction; HFpEF=Heart failure with preserved ejection fraction. | | | | | | |
